# Supplementary material for: A Proteomic Study of the Bioactivity of Annona muricata Leaf Extracts in HT-1080 Fibrosarcoma Cells
Source: Int J Mol Sci. 2023 Jul 27;24(15):12021. doi: 10.3390/ijms241512021 (PMC10418445; doi:10.3390/ijms241512021)
Supplement: Supplementary file 1 [file ijms-24-12021-s001.zip › ijms-2447024-supplementary.pdf]

Figure S1. Network stats and functional enrichment details.

Network Stats

number of nodes:	29	expected number of edges:	3
number of edges:	7	PPI enrichment p-value:	0.0207
average node degree:	0.483		
avg. local clustering coefficient:	0.253		

Functional enrichments

	Biological Process (Gene Ontology)			
GO-term	*description*	*count in network*	*strength*	*false discovery rate*
GO:0010032	Meiotic chromosome condensation	3 of 7	2.46	0.0045
GO:0007076	Mitotic chromosome condensation	3 of 16	2.1	0.0179

	Cellular Component (Gene Ontology)			
GO-term	*description*	*count in network*	*strength*	*false discovery rate*
GO:0000797	Condensin core heterodimer	3 of 3	2.83	9.87e-05
GO:0000799	Nuclear condensin complex	2 of 4	2.53	0.0179

	Subcellular localization (COMPARTMENTS)			
compartment	*description*	*count in network*	*strength*	*false discovery rate*
GOCC:0000797	Condensin core heterodimer	2 of 2	2.83	0.0098
GOCC:0061814	Condensin I complex	2 of 6	2.35	0.0227
GOCC:0005634	Nucleus	17 of 4636	0.39	0.0227

	Annotated Keywords (UniProt)			
keyword	*description*	*count in network*	*strength*	*false discovery rate*
KW-0444	Lipid biosynthesis	4 of 163	1.22	0.0346

For STRING analysis, the minimum required interaction score has been 0.7 (high confidence). The adjacent tables show a network statistics reporting data concerning the number of nodes and edges, the average node degree, the average local clustering coefficient, the expected number of edges, and the protein-protein interaction (PPI) enrichment p-value. Functional enrichment table indicates: (i) Count In Network: the first number indicates how many proteins in your network are annotated with a particular term. The second number indicates how many proteins in total in your network and in the whole genome have this term assigned. (ii) Strength: Log10(observed/expected). The p-values corrected for multiple testing within each category using the Benjamini-Hochberg proce-dure are shown.

Figure S2. ***Annona muricata*** **extracts do not induce ferroptosis**. Representative bright field and fluorescence images of HT1080 treated only with *Annona muricata* DMSO or aqueous extracts, as negative controls of induction of ferroptosis.

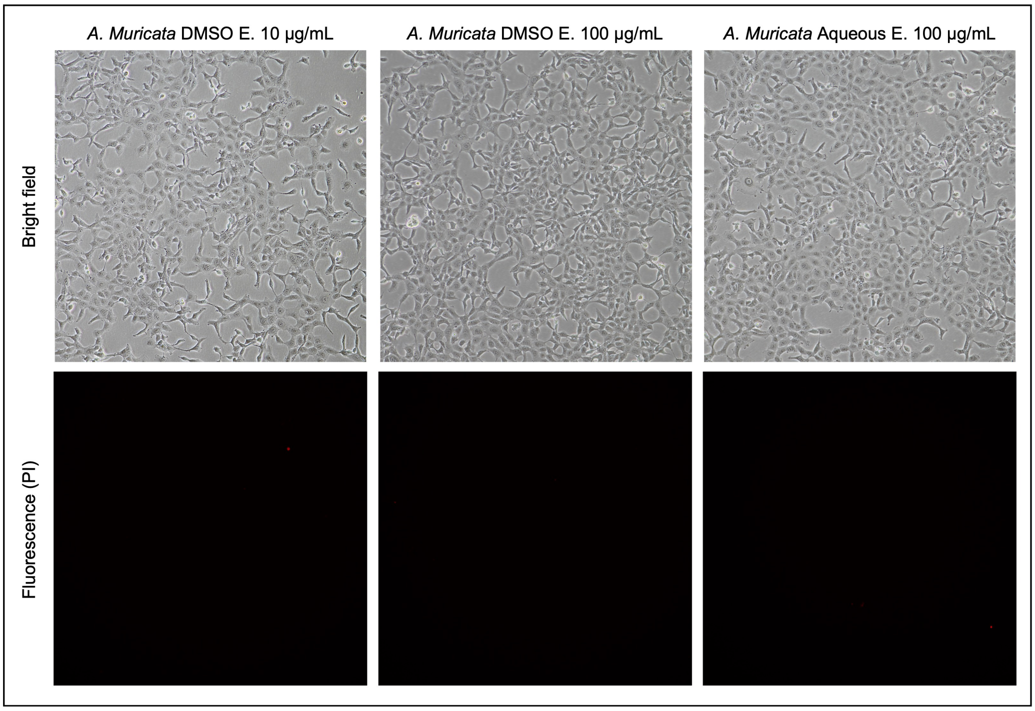

Table S1. Significantly over- and underexpressed proteins in HT1080 cell extracts after treatment with aqueous extract from *Annona muricata.*

Gene Symbol	UniProt Accession	Description	Sum PEP score*	Abundance Ratio: Treatment/Control	Abundance Ratio P-Value
HRNR	Q86YZ3	Hornerin [OS=Homo sapiens]	41,25	5,61	0,04728
PSMD9	O00233	26S proteasome non-ATPase regulatory subunit 9 [OS=Homo sapiens]	19,60	5,04	0,02041
NUCB2	P80303	Nucleobindin-2 [OS=Homo sapiens]	8,64	4,47	0,02511
RALB	P11234	Ras-related protein Ral-B [OS=Homo sapiens]	13,99	3,91	0,03944
HTATSF1	O43719	HIV Tat-specific factor 1 [OS=Homo sapiens]	5,43	3,88	0,00092
SMTN	P53814	Smoothelin [OS=Homo sapiens]	6,82	3,42	0,04812
RPL35A	P18077	60S ribosomal protein L35a [OS=Homo sapiens]	25,15	2,95	0,00250
ATP5IF1	Q9UII2	ATPase inhibitor, mitochondrial [OS=Homo sapiens]	4,02	2,82	0,02375
COL4A2	P08572	Collagen alpha-2(IV) chain [OS=Homo sapiens]	17,95	2,58	0,00661
AHCYL1	O43865	S-adenosylhomocysteine hydrolase-like protein 1 [OS=Homo sapiens]	21,00	2,57	0,00207
	Q9NX58	Cell growth-regulating nucleolar protein [OS=Homo sapiens]	9,55	2,51	0,03535
S100A16	Q96FQ6	Protein S100-A16 [OS=Homo sapiens]	16,70	2,47	0,00114
STC1	P52823	Stanniocalcin-1 [OS=Homo sapiens]	10,59	2,39	0,04756
HK2	P52789	Hexokinase-2 [OS=Homo sapiens]	20,13	2,39	0,02960
BAG5	Q9UL15	BAG family molecular chaperone regulator 5 [OS=Homo sapiens]	4,03	2,38	0,04229
RPS28	P62857	40S ribosomal protein S28 [OS=Homo sapiens]	8,01	2,37	0,01055
PPP1R2B	Q6NXS1	Protein phosphatase inhibitor 2 family member B [OS=Homo sapiens]	6,28	2,36	0,00214

GNG12	Q9UBI6	Guanine nucleotide-binding protein G(I)/G(S)/G(O) subunit gamma-12 [OS=Homo sapiens]	12,00	2,31	0,01577
SNRPC	P09234	U1 small nuclear ribonucleoprotein C [OS=Homo sapiens]	7,42	2,27	0,01755
PPIH	O43447	Peptidyl-prolyl cis-trans isomerase H [OS=Homo sapiens]	10,90	2,23	0,00150
SMARCE1	Q969G3	SWI/SNF-related matrix-associated actin-dependent regulator of chromatin subfamily E member 1 [OS=Homo sapiens]	7,09	2,22	0,04804
CLTB	P09497	Clathrin light chain B [OS=Homo sapiens]	9,56	2,17	0,01074
STMN1	P16949	Stathmin [OS=Homo sapiens]	21,27	2,17	0,01049
BCAR1	P56945	Breast cancer anti-estrogen resistance protein 1 [OS=Homo sapiens]	18,19	2,13	0,03699
PURB	Q96QR8	Transcriptional activator protein Pur-beta [OS=Homo sapiens]	19,61	2,11	0,00134
EIF4B	P23588	Eukaryotic translation initiation factor 4B [OS=Homo sapiens]	82,28	2,06	0,01606
RPS17	P08708	40S ribosomal protein S17 [OS=Homo sapiens]	36,77	2,05	0,01888
COX5B	P10606	Cytochrome c oxidase subunit 5B, mitochondrial [OS=Homo sapiens]	18,89	2,05	0,01385
S100A6	P06703	Protein S100-A6 [OS=Homo sapiens]	7,35	2,05	0,04501
TFRC	P02786	Transferrin receptor protein 1 [OS=Homo sapiens]	91,93	2,02	0,00087
GRIPAP1	Q4V328	GRIP1-associated protein 1 [OS=Homo sapiens]	4,27	2,01	0,03573
PCNP	Q8WW12	PEST proteolytic signal-containing nuclear protein [OS=Homo sapiens]	16,90	2,00	0,01940
NIF3L1	Q9GZT8	NIF3-like protein 1 [OS=Homo sapiens]	13,93	0,50	0,01253
WARS1	P23381	Tryptophan--tRNA ligase, cytoplasmic [OS=Homo sapiens]	75,70	0,49	0,02655

EIF2B2	P49770	Translation initiation factor eIF-2B subunit beta [OS=Homo sapiens]	16,29	0,48	0,04503
RPAP3	Q9H6T3	RNA polymerase II-associated protein 3 [OS=Homo sapiens]	10,51	0,48	0,00499
LACTB	P83111	Serine beta-lactamase-like protein LACTB, mitochondrial [OS=Homo sapiens]	8,74	0,47	0,00207
CUL2	Q13617	Cullin-2 [OS=Homo sapiens]	33,84	0,47	0,00254
SMU1	Q2TAY7	WD40 repeat-containing protein SMU1 [OS=Homo sapiens]	18,33	0,46	0,01453
POLDIP2	Q9Y2S7	Polymerase delta-interacting protein 2 [OS=Homo sapiens]	13,00	0,46	0,01877
AXL	P30530	Tyrosine-protein kinase receptor UFO [OS=Homo sapiens]	8,93	0,45	0,00997
EIF3D	O15371	Eukaryotic translation initiation factor 3 subunit D [OS=Homo sapiens]	43,15	0,45	0,00111
PPP2R1B	P30154	Serine/threonine-protein phosphatase 2A 65 kDa regulatory subunit A beta isoform [OS=Homo sapiens]	18,22	0,45	0,01004
DYNLL1	P63167	Dynein light chain 1, cytoplasmic [OS=Homo sapiens]	18,53	0,45	0,00182
SLC20A1	Q8WUM9	Sodium-dependent phosphate transporter 1 [OS=Homo sapiens]	10,75	0,44	0,00768
TYMS	P04818	Thymidylate synthase [OS=Homo sapiens]	19,39	0,44	0,01364
RAB27B	O00194	Ras-related protein Rab-27B [OS=Homo sapiens]	16,14	0,44	0,00371
PRRC2A	P48634	Protein PRRC2A [OS=Homo sapiens]	13,89	0,43	0,03776
MRPS16	Q9Y3D3	28S ribosomal protein S16, mitochondrial [OS=Homo sapiens]	5,31	0,43	0,00397
	Q16850	Lanosterol 14-alpha demethylase [OS=Homo sapiens]	32,21	0,43	0,00078
PPME1	Q9Y570	Protein phosphatase methylesterase 1 [OS=Homo sapiens]	9,37	0,41	0,02185

PDP1	Q9P0J1	[Pyruvate dehydrogenase [acetyl-transferring]]-phosphatase 1, mitochondrial [OS=Homo sapiens]	41,19	0,40	0,00348
NDUFS3	O75489	NADH dehydrogenase [ubiquinone] iron-sulfur protein 3, mitochondrial [OS=Homo sapiens]	26,21	0,40	0,02990
KDM1A	O60341	Lysine-specific histone demethylase 1A [OS=Homo sapiens]	11,52	0,39	0,00135
BLMH	Q13867	Bleomycin hydrolase [OS=Homo sapiens]	20,12	0,39	0,00111
NCAPD2	Q15021	Condensin complex subunit 1 [OS=Homo sapiens]	7,70	0,39	0,02709
ADI1	Q9BV57	1,2-dihydroxy-3-keto-5-methylthiopentene dioxygenase [OS=Homo sapiens]	12,31	0,39	0,02647
KDSR	Q06136	3-ketodihydrosphingosine reductase [OS=Homo sapiens]	6,79	0,38	0,00934
MYEF2	Q9P2K5	Myelin expression factor 2 [OS=Homo sapiens]	9,70	0,38	0,00447
MVK	Q03426	Mevalonate kinase [OS=Homo sapiens]	17,79	0,38	0,01204
EXOSC2	Q13868	Exosome complex component RRP4 [OS=Homo sapiens]	16,72	0,37	0,00136
FASTKD2	Q9NYY8	FAST kinase domain-containing protein 2, mitochondrial [OS=Homo sapiens]	6,20	0,37	0,00468
SLC39A14	Q15043	Metal cation symporter ZIP14 [OS=Homo sapiens]	9,83	0,35	0,00105
SPTBN2	O15020	Spectrin beta chain, non-erythrocytic 2 [OS=Homo sapiens]	14,15	0,34	0,03169
BCAP29	Q9UHQ4	B-cell receptor-associated protein 29 [OS=Homo sapiens]	9,85	0,33	0,02658
NOC4L	Q9BVI4	Nucleolar complex protein 4 homolog [OS=Homo sapiens]	5,28	0,33	0,03488
HAT1	O14929	Histone acetyltransferase type B catalytic subunit [OS=Homo sapiens]	14,64	0,31	0,01274

FTH1	P02794	Ferritin heavy chain [OS=Homo sapiens]	22,66	0,26	0,00028
PPAT	Q06203	Amidophosphoribosyltransferase [OS=Homo sapiens]	15,53	0,26	0,00205
UACA	Q9BZF9	Uveal autoantigen with coiled-coil domains and ankyrin repeats [OS=Homo sapiens]	5,70	0,24	0,01286
CDH2	P19022	Cadherin-2 [OS=Homo sapiens]	7,21	0,23	0,02434
MAPK9	P45984	Mitogen-activated protein kinase 9 [OS=Homo sapiens]	4,86	0,22	0,00203
ESF1	Q9H501	ESF1 homolog [OS=Homo sapiens]	7,14	0,22	0,00001
UBA5	Q9GZZ9	Ubiquitin-like modifier-activating enzyme 5 [OS=Homo sapiens]	10,44	0,21	0,00201
PIGS	Q96S52	GPI transamidase component PIG-S [OS=Homo sapiens]	5,77	0,21	0,02276
THBS1	P07996	Thrombospondin-1 [OS=Homo sapiens]	38,46	0,20	0,00228
AATF	Q9NY61	Protein AATF [OS=Homo sapiens]	10,13	0,18	0,04332
FADS2	O95864	Acyl-CoA 6-desaturase [OS=Homo sapiens]	34,42	0,18	0,01124
FAM98A	Q8NCA5	Protein FAM98A [OS=Homo sapiens]	13,98	0,16	0,04574
PITRM1	Q5JRX3	Presequence protease, mitochondrial [OS=Homo sapiens]	6,60	0,10	0,00192
CEMIP2	Q9UHN6	Cell surface hyaluronidase [OS=Homo sapiens]	7,00	0,06	0,00016

*The sum PEP score corresponds to the score calculated based on the posterior error probability (PEP) values of the peptide spectrum matches (PSM). The PEP indicates the probability that an observed PSM is a random event. Sum PEP score is calculated as the negative logarithms of the PEP values of the connected PSM.

Table S2. Significantly over- and underexpressed proteins in HT1080 cell extracts after treatment with DMSO extract from *Annona muricata.*					
**Gene Symbol**	**UniProt Accession**	**Description**	**Sum PEP score***	**Abundance Ratio: Treatment/Control**	**Abundance Ratio P-Value**
WDR26	Q9H7D7	WD repeat-containing protein 26 [OS=Homo sapiens]	10,82	19,30	0,00046
RALB	P11234	Ras-related protein Ral-B [OS=Homo sapiens]	11,74	3,41	0,02986
SLC7A11	Q9UPY5	Cystine/glutamate transporter [OS=Homo sapiens]	5,22	3,01	0,04369
CHCHD2P9	Q5T1J5	Putative coiled-coil-helix-coiled-coil-helix domain-containing protein CHCHD2P9, mitochondrial [OS=Homo sapiens]	8,09	2,68	0,02272
MRPS5	P82675	28S ribosomal protein S5, mitochondrial [OS=Homo sapiens]	10,09	2,44	0,01895
HRNR	Q86YZ3	Hornerin [OS=Homo sapiens]	28,83	2,43	0,04102
SAAL1	Q96ER3	Protein SAAL1 [OS=Homo sapiens]	5,85	2,33	0,01740
GOLPH3	Q9H4A6	Golgi phosphoprotein 3 [OS=Homo sapiens]	7,98	2,30	0,04552
	Q9NX58	Cell growth-regulating nucleolar protein [OS=Homo sapiens]	14,48	2,27	0,03416
ASPSCR1	Q9BZE9	Tether containing UBX domain for GLUT4 [OS=Homo sapiens]	5,38	2,20	0,03274
H1-10	Q92522	Histone H1.10 [OS=Homo sapiens]	17,93	2,17	0,00512
UPP1	Q16831	Uridine phosphorylase 1 [OS=Homo sapiens]	5,85	2,14	0,02702
SREK1	Q8WXA9	Splicing regulatory glutamine/lysine-rich protein 1 [OS=Homo sapiens]	15,75	2,10	0,03789
SNX5	Q9Y5X3	Sorting nexin-5 [OS=Homo sapiens]	21,40	2,09	0,03354
SLC2A3	P11169	Solute carrier family 2, facilitated glucose transporter member 3 [OS=Homo sapiens]	10,56	2,06	0,04256
SMC2	O95347	Structural maintenance of chromosomes protein 2 [OS=Homo sapiens]	50,27	0,50	0,01179

EIF2B4	Q9UI10	Translation initiation factor eIF-2B subunit delta [OS=Homo sapiens]	6,19	0,49	0,00250
ANKRD28	O15084	Serine/threonine-protein phosphatase 6 regulatory ankyrin repeat subunit A [OS=Homo sapiens]	8,69	0,49	0,01278
THBS1	P07996	Thrombospondin-1 [OS=Homo sapiens]	38,49	0,47	0,00775
NOSIP	Q9Y314	Nitric oxide synthase-interacting protein [OS=Homo sapiens]	15,77	0,47	0,02365
PPP2R1B	P30154	Serine/threonine-protein phosphatase 2A 65 kDa regulatory subunit A beta isoform [OS=Homo sapiens]	18,75	0,46	0,01657
SPARC	P09486	SPARC [OS=Homo sapiens]	12,17	0,46	0,00498
MYEF2	Q9P2K5	Myelin expression factor 2 [OS=Homo sapiens]	9,60	0,45	0,00547
FADS1	O60427	Acyl-CoA (8-3)-desaturase [OS=Homo sapiens]	7,29	0,45	0,01636
KPNA2	P52292	Importin subunit alpha-1 [OS=Homo sapiens]	39,57	0,45	0,01288
SRRM1	Q8IYB3	Serine/arginine repetitive matrix protein 1 [OS=Homo sapiens]	16,12	0,45	0,02866
HAT1	O14929	Histone acetyltransferase type B catalytic subunit [OS=Homo sapiens]	15,76	0,45	0,00559
SMC4	Q9NTJ3	Structural maintenance of chromosomes protein 4 [OS=Homo sapiens]	41,05	0,43	0,00510
RAB3GAP 1	Q15042	Rab3 GTPase-activating protein catalytic subunit [OS=Homo sapiens]	5,27	0,42	0,02826
CSNK1A1	P48729	Casein kinase I isoform alpha [OS=Homo sapiens]	8,56	0,41	0,01953
MVK	Q03426	Mevalonate kinase [OS=Homo sapiens]	17,65	0,40	0,00916
CC2D1B	Q5T0F9	Coiled-coil and C2 domain-containing protein 1B [OS=Homo sapiens]	5,75	0,39	0,00035
FAM98A	Q8NCA5	Protein FAM98A [OS=Homo sapiens]	13,47	0,38	0,03535
	Q16850	Lanosterol 14-alpha demethylase [OS=Homo sapiens]	31,95	0,36	0,00639

CHD4	Q14839	Chromodomain-helicase-DNA-binding protein 4 [OS=Homo sapiens]	8,98	0,36	0,04954
FADS2	O95864	Acyl-CoA 6-desaturase [OS=Homo sapiens]	35,16	0,35	0,00546
DIDO1	Q9BTC0	Death-inducer obliterator 1 [OS=Homo sapiens]	5,28	0,34	0,00257
NCAPD2	Q15021	Condensin complex subunit 1 [OS=Homo sapiens]	9,25	0,31	0,00074
CD63	P08962	CD63 antigen [OS=Homo sapiens]	7,09	0,30	0,00134
PLSCR1	O15162	Phospholipid scramblase 1 [OS=Homo sapiens]	4,51	0,25	0,02525
PRRC2A	P48634	Protein PRRC2A [OS=Homo sapiens]	13,90	0,23	0,00973
CDK4	P11802	Cyclin-dependent kinase 4 [OS=Homo sapiens]	8,95	0,20	0,01429
HNRNPLL	Q8WVV9	Heterogeneous nuclear ribonucleoprotein L-like [OS=Homo sapiens]	4,61	0,18	0,00573
RUNX1	Q01196	Runt-related transcription factor 1 [OS=Homo sapiens]	7,27	0,07	0,00663
IGHM	P01871	Immunoglobulin heavy constant mu [OS=Homo sapiens]	52,10	0,02	0,01076

*The sum PEP score corresponds to the score calculated based on the posterior error probability (PEP) values of the peptide spectrum matches (PSM). The PEP indicates the probability that an observed PSM is a random event. Sum PEP score is calculated as the negative logarithms of the PEP values of the connected PSM.

Table S3. Significantly over- and underexpressed proteins in HT1080 culture media after treatment with aqueous extract from *Annona muricata.*

Gene Symbol	UniProt Accession	Description	Sum PEP score*	Abundance Ratio: Treatment/Control	Abundance Ratio P-Value
SEMA3A	Q14563	Semaphorin-3A [OS=Homo sapiens]	14,22	4,55	0,01135
LRRC17	Q8N6Y2	Leucine-rich repeat-containing protein 17 [OS=Homo sapiens]	14,30	3,65	0,00028
SEMA3C	Q99985	Semaphorin-3C [OS=Homo sapiens]	42,14	3,51	0,03081
DIPK2A	Q8NDZ4	Divergent protein kinase domain 2A [OS=Homo sapiens]	8,83	2,76	0,01231
GDF15	Q99988	Growth/differentiation factor 15 [OS=Homo sapiens]	164,70	2,69	0,00044
HHIP	Q96QV1	Hedgehog-interacting protein [OS=Homo sapiens]	79,59	2,52	0,00002
THSD4	Q6ZMP0	Thrombospondin type-1 domain-containing protein 4 [OS=Homo sapiens]	58,47	2,46	0,00439
GLG1	Q92896	Golgi apparatus protein 1 [OS=Homo sapiens]	94,17	2,18	0,00222
CEMIP	Q8WUJ3	Cell migration-inducing and hyaluronan-binding protein [OS=Homo sapiens]	241,80	2,07	0,00078
S100A8	P05109	Protein S100-A8 [OS=Homo sapiens]	12,49	0,42	0,02702
AZGP1	P25311	Zinc-alpha-2-glycoprotein [OS=Homo sapiens]	9,70	0,41	0,04529
FTH1	P02794	Ferritin heavy chain [OS=Homo sapiens]	24,01	0,35	0,00644
H4C_	P62805	Histone H4 [OS=Homo sapiens]	101,56	0,29	0,04128
H3-3A; H3-3B	P84243	Histone H3.3 [OS=Homo sapiens]	17,46	0,27	0,00124
FTL	P02792	Ferritin light chain [OS=Homo sapiens]	20,99	0,21	0,01911

*The sum PEP score corresponds to the score calculated based on the posterior error probability (PEP) values of the peptide spectrum matches (PSM). The PEP indicates the probability that an observed PSM is a random event. Sum PEP score is calculated as the negative logarithms of the PEP values of the connected PSM.

Table S4. Significantly over- and underexpressed proteins in HT1080 culture media after treatment with DMSO extract from *Annona muricata.*

**Gene Symbol**	**UniProt Accession**	**Description**	**Sum PEP score***	**Abundance Ratio: Treatment/Control**	**Abundance Ratio P-Value**
ERAP1	Q9NZ08	Endoplasmic reticulum aminopeptidase 1 [OS=Homo sapiens]	86,35	4,38	0,00399
FAU	P62861	40S ribosomal protein S30 [OS=Homo sapiens]	11,62	3,57	0,02734
YBX1	P67809	Y-box-binding protein 1 [OS=Homo sapiens]	118,34	2,18	0,02061
TNFSF9	P41273	Tumor necrosis factor ligand superfamily member 9 [OS=Homo sapiens]	7,56	0,49	0,03410
CHID1	Q9BWS9	Chitinase domain-containing protein 1 [OS=Homo sapiens]	20,03	0,43	0,03206
CALU	O43852	Calumenin [OS=Homo sapiens]	76,54	0,43	0,00473
TSKU	Q8WUA8	Tsukushi [OS=Homo sapiens]	29,73	0,39	0,02649
NOG	Q13253	Noggin [OS=Homo sapiens]	45,75	0,27	0,04310

*The sum PEP score corresponds to the score calculated based on the posterior error probability (PEP) values of the peptide spectrum matches (PSM). The PEP indicates the probability that an observed PSM is a random event. Sum PEP score is calculated as the negative logarithms of the PEP values of the connected PSM.

Table S5. List of bio-active compounds previously identified by us in both aqueous and DMSO *Annona muricata* leaf extracts.

**Aqueous extract**	**DMSO extract**
Annoionoside	Anonaine
Argentinine	Chlorogenic acid
Catechin	Coclaurine
Chlorogenic acid	Corossolone
Isolaureline	Isolaureline
Isoquercetin	Isoquercetin
Isoquercetin	Kaempferol
Kaempferol	Loliolide
Kaempferol-3-*O*-rutinoside	Norcorydine
Loliolide	Quercetin
Norcorydine	Reticuline
Quercetin	Rutin
Reticuline	Xylopine
Rutin	
Stepharine	
Vomifoliol	

These compounds were identified by UHPLC-HRMS according to the procedures described in [7].
